# Trophic diversity in two grassland ecosystems

**DOI:** 10.1673/2006_06_25.1

**Published:** 2006-09-28

**Authors:** Clark V. Pearson, Lee A. Dyer

**Affiliations:** Department of Ecology and Evolutionary Biology, Tulane University, New Orleans, LA. 70118

**Keywords:** arthropods, grazing, diversity cascades, community ecology

## Abstract

The roles of consumers (top-down forces) versus resources (bottom-up forces) as determinants of alpha diversity in a community are not well studied. Numerous community ecology models and empirical studies have provided a framework for understanding how density at various trophic levels responds to variation in the relative strength of top-down and bottom-up forces. The resulting trophic theory can be applied to understanding variation in insect diversity at different trophic levels. The objective of this research was to elucidate the strengths of direct and indirect interactions between plants and entire arthropod communities to determine the effects of trophic interactions on arthropod diversity. Grassland plant and insect diversity was measured in July 2001 to document patterns of diversity at multiple trophic levels. The study site includes riparian grasslands in North-Central Colorado on the Carpenter Ranch, owned and managed by The Nature Conservancy. This pastureland consists of sites with different management regimes: unmanaged pasture intermixed along riparian forest, and cattle grazed pasture with flood irrigation. Plant abundance and richness were higher on the grazed-irrigated pasture versus the unmanaged field. Path analysis revealed strong effects of herbivore diversity on diversity of other trophic levels. For the managed fields, top-down forces were important, with increases in enemy diversity depressing herbivore diversity, which in turn depressed plant abundance. For the unmanaged fields, bottom-up forces dominated, with increases in plant diversity causing increased herbivore diversity, which in turn increased enemy diversity. These results support hypotheses from other empirical studies, demonstrating that changes in diversity of a single trophic level can cascade to effect diversity at other, nonadjacent trophic levels.

## Introduction

Deciphering the factors that determine diversity and number of trophic levels in a community, or “community structure,” is a major goal of community ecology. Since the publication of Hairston et al’s (1960) hypothesis that detritivores, plants and predators are resource limited while herbivores are consumer limited, a number of theoretical and empirical studies have examined the roles of trophic interactions in determining community structure (reviewed by Dyer et al. 1993; Pace et al. 1999; Persson 1999; Polis 1999;Holt 2000; Halaj and Wise 2001; Shurin et al. 2002; Chase 2003; Stireman et al. 2005). Currently, focus has shifted away from the dichotomous view of the importance of top-down (natural enemies and herbivores) versus bottom-up (plant quality and abundance) forces to a more synthetic view on how these factors may act in concert to regulate communities (Oksanen 1991; Leibold 1996; Dunne et al. 2002).

Because of the complexity found in terrestrial systems, including intraguild predation, omnivory, and ontogenetic diet changes, it has been suggested that the concept of trophic levels is of no use to terrestrial community ecologists (Polis and Strong 1996;Hunter 2001). However, work by Schmitz and Sokol-Hessner (2002) and by others demonstrates that at least some terrestrial systems exhibit a strong trophic structure (reviewed by Schmitz et al. 2000). Even complex, reticulate communities can be examined with the assumption that trophic levels exist, and this concept has long been useful for community ecologists studying dynamics among various species. Instead of abandoning trophic level concepts for terrestrial systems, more empirical tests are needed to determine the roles of omnivory, intraguild predation, life history omnivores and their effects on mediating top-down and bottom-up forces.

Factors that maintain alpha diversity within a particular trophic level have been well studied (e.g., Tilman 1982; Wright 2002; Sax and Gaines 2003), but little consensus has been reached on the most important biotic determinants of alpha diversity, in large part because of the number of mechanisms proposed, a paucity of empirical tests, and confounding effects of multiple mechanisms making isolation of any one unlikely (Wright 2002). Also, there are surprisingly few experiments in which an entire community has been examined with diversity as the response variable (Carson and Root 2000; Dyer and Letourneau 2003;Dyer and Stireman 2003). As a result, little is known about interactions between trophic level diversity at multiple levels in any given community. While many studies investigate the role that specific predators have on lower trophic levels, few studies investigate the dynamics of how consumer diversity affects resource diversity along a trophic chain and vice versa.

Theoretical and empirical studies of ‘diversity cascades’ (defined as an indirect effect of diversity at one trophic level on a non-adjacent trophic level, sensuDyer and Letourneau 2003) are controversial and not well understood. One established paradigm is that the diversity of enemies and herbivores will increase with primary productivity (Hutchinson 1959;Huston 1994; Abrams et al. 1995; Siemann 1998; Srivastava and Lawton 1998). However, there are exceptions to this hypothesized relationship, with many studies demonstrating no effect or even results that are in the opposite direction from predictions (Rosenzweig and Abramsky 1993; Waide et al. 1999; Kassan, et al. 2000;Mittelbach et al 2001; Otway et al. 2005). Community ecologists are still far from synthesizing these results into general theory, and while empirical studies focus on short-term experiments, realistic correlational studies are also necessary for a clearer picture of interactions between diversity at multiple trophic levels (Leibold et al. 1997).

The goal of our research was to investigate how trophic interactions affect alpha diversity in a community consisting of grasslands and associated arthropods, including herbivores, omnivores, predators and parasitoids. In an experimental study in nearby alfalfa fields in Colorado, Dyer and Stireman (2003) found that management (irrigation, application of pesticides, and experimental removal of arthropods) altered trophic interactions in alfalfa fields in Colorado. Alfalfa fields with experimentally depleted arthropod richness exhibited the strongest bottom up effects, with enhanced plant resources causing increased abundance and diversity of upper trophic levels. In contrast, for fields that had higher diversity of natural enemies due to management, top-down effects of increased enemy diversity on lower trophic levels were more important (Dyer and Stireman 2003). These results were consistent with a number of studies that have utilized experiments and path analysis to examine diversity relationships between trophic levels and with studies of biological control. In agricultural or experimental systems, where plant diversity is relatively low (compared to wild fields), studies have demonstrated that increases in plant diversity enhance overall arthropod diversity (Siemann et al. 1998;Koricheva et al. 2000), although this is not universal (Koricheva et al. 2000). It is possible that the studies that failed to demonstrate bottom up cascades on diversity are in communities where top-down effects are stronger, such as the enemy-diverse alfalfa fields studied by Dyer and Stireman (2003).

A paradigm in biological control is that increases in predator diversity weaken the effects of natural enemies via intraguild predation and omnivory (Hochberg 1996; Denoth et al. 2002). However, when overall community diversity is increased, including higher plant diversity, the “enemies hypothesis” (Root 1973) predicts stronger top-down effects of enemies on herbivores. In addition, a number of researchers have recently argued that a diverse natural enemy fauna may often result in more effective regulation of prey populations and stronger positive effects on primary producers (e.g., Reichert and Lawrence 1997; Losey and Denno 1998, 1999; Cardinale et al. 2003). These patterns are not well supported by empirical data in natural or managed systems, therefore it is appropriate to test hypotheses that top-down diversity cascades occur in more diverse communities, and that this flips to bottom-up cascades as overall plant and arthropod diversity decreases. Based on the diversity patterns described above and corroborating results from nearby alfalfa fields (Dyer and Stireman 2003), we tested the following hypotheses: 1) Management for grazing in the focal grasslands is sufficient to generate strong differences in plant and arthropod diversity and on trophic interactions. 2) In fields with lower plant and arthropod richness, resource availability directly enhances herbivore diversity and indirectly enhances diversity of upper trophic levels. 3) In fields with higher diversity of enemies, increases in natural enemy diversity will cause decreases in herbivore diversity and subsequent changes in herbivore density and plant abundance. Correlational field data were collected and path analysis was utilized to test these specific hypotheses.

## Methods and Materials

This study took place on Nature Conservancy property, Carpenter Ranch, in North-Central Colorado (1,950 meters, 40.50° N 107.16° W). Two types of field communities were sampled: grazed and irrigated cattle pasture (hereafter referred to as managed fields), and ungrazed, protected fields interspersed within the riparian gallery forest of the Yampa River (hereafter referred to as unmanaged fields). All fields occur within the floodplain of the river and are composed of alluvial soils and cobblestones at variable depths. The dominant vegetation in the unmanaged fields is grass, primarily Bromus inermis (Poaceae). Forage grasses and clover are most abundant in the managed fields (See Appendix A for common families of plants and species richness).

In July 2001, arthropods were sampled in two distinct ecosystems at the study area (see Appendix C). Four sites were chosen, with two fields per management type; Upper and Lower Marshall (UM & LM), Upper Marshall Buffer and Hein Island (UMB & HI). Twenty rectangular plots (1 x 1.5m) that were uniform in slope and drainage were established in each field, and were marked with bright orange rebar safety caps set flush with the ground in the North-West corner in each plot. Each plot was aligned with the long side (1.5m) on a North-South axis. In each plot, all plant species were identified and the number of shoots per species was recorded. As each new species of plant was encountered, a specimen was collected from outside the plot and preserved for later identification.

To sample arthropods, a gasoline powered, reversible leaf blower was used in which a fabric bag was securely inserted into the vacuum end, capturing insects and debris. This “insect vac” was passed over the plot for 20–40 seconds, or until no insect activity was detected. When finished with each plot the specimens were removed from the bag and emptied into a one-gallon plastic food bag that contained a cotton ball soaked with ethyl acetate to kill the specimens. The samples were stored briefly in a freezer to ensure the specimens were dead and then sent to Tulane University for identification.

Arthropod samples were sorted to morphospecies, identified to the lowest taxonomic level possible, and assigned a feeding level according to published literature and discussion with taxonomists. Morphospecies were assigned to a general trophic group: detritivore, herbivore, predator, parasitoid, or omnivore. The Shannon-Weiner index of diversity (H') was calculated for the entire food web (plants & arthropods) and for different trophic levels. Detritivores, mostly collembolans, were not counted in all the plots because of their overwhelming abundance. The omnivore category included specimens that were not identified to a sufficient level (mostly muscoid flies). Because the quality of the data was insufficient for these guilds, detritivores and omnivores were not included in analyses of trophic interactions. Up to twenty individuals from each morphospecies were preserved according to standard entomological protocol and entered into voucher collections at Tulane University and at Carpenter Ranch.

**Figure 1:**
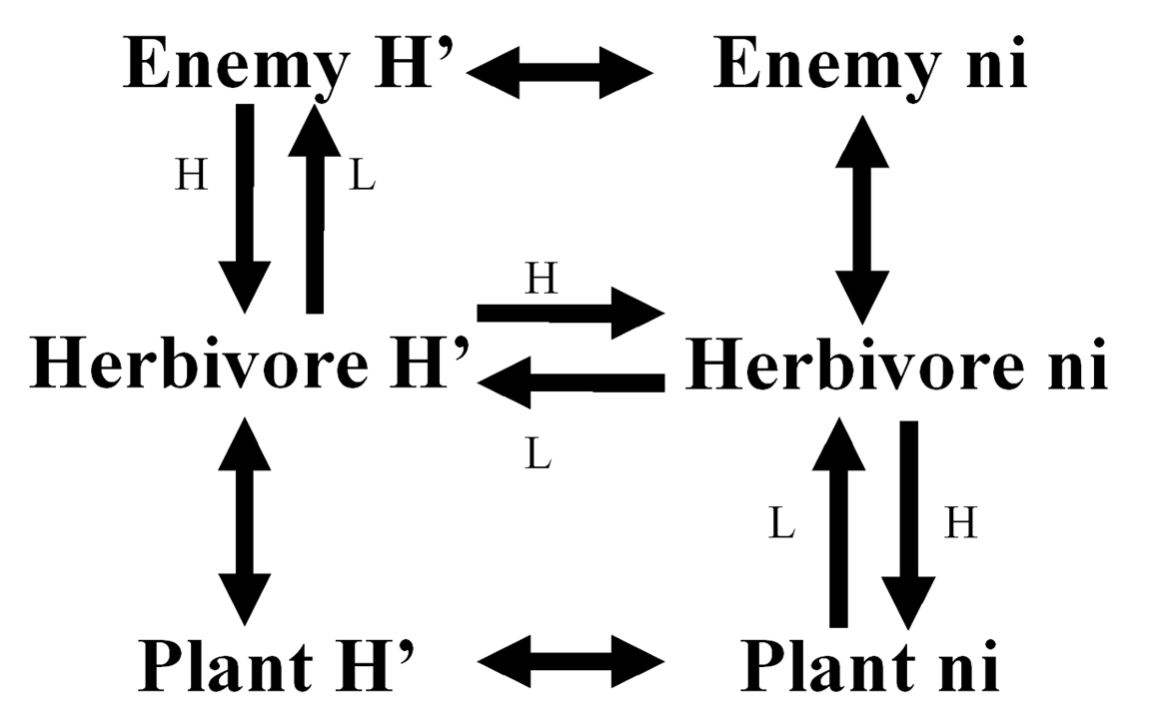
Path diagram with predicted trophic relationships based on previous studies. Single-headed arrows indicate hypothesized causal relationships, double-headed arrows indicate correlations; arrows do not indicate type of effect (positive versus negative). “L” pathway includes predicted causal relationships in low diversity fields; “H” pathway includes predicted causal relationships in high diversity fields. Correlations between other variables are also predicted to be important in both types of fields. H’ - Shannon-Weiner Diversity Index; ni – abundance.

Species accumulation curves and estimates of species richness were calculated using ‘Estimate S’ (Colwell 2004), with 50 random samples for estimation of means and standard errors. The Chao 1 estimator of species richness was calculated, which is appropriate for studies with uniform sample size and collection method. The diversity (H') values calculated for trophic levels in replicated plots were used to test specific path models, derived from previous structural equation models of diversity cascades in nearby agricultural systems (Dyer and Stireman 2003) (Fig. 1). Structural equation models and path analysis are used with multiple regression to examine proposed causal pathways between variables that have been measured in correlational or experimental studies (Shipley 2000). A specific pathway, such as “increased plant biomass causes increased herbivore biomass, which in turn causes an increase in enemy diversity,” suggests a very specific correlational matrix that is statistically distinct from a pathway that assumes correlations between all of these variables (e.g., plant biomass, herbivore biomass, enemy diversity). For example, the partial correlation between plant biomass and enemy diversity that controls for herbivore biomass should not be significant if the hypothesized plant-herbivore-enemy causal pathway is correct. Like experimental approaches, this approach relies on controlling one variable, while allowing others to vary, but with path analysis the control is statistical rather than physical (Shipley 2000). Structural equation modeling allows for statistical comparisons between different causal and correlational pathways.

Used appropriately and in conjunction with experiments, path analysis can be a powerful tool in investigating causal pathways and determining the magnitude of interactions between variables. In addition, the benefit of utilizing path diagrams is that abundant information can be displayed, summarizing complex correlational matrices and causal pathways. The path models tested with our correlational data are displayed in Figure 1. For a localized comparison of management strategies, simple linear regression analysis was utilized for all diversity variables, with field type (managed versus unmanaged) as an independent variable. Plots within each management type were pseudoreplicates, but the analyses were only conducted to compare these specific fields and not to generalize about management type. Calculation of diversity indices, regression analysis and path analysis was performed using SAS statistical software (SAS 8.0).

## Results

There were 267 arthropod morpho-species collected from all plots, with more than 50 families and 12 orders represented for a total of 25,453 individuals. The dominant orders (based on both species richness and abundance) were Hymenoptera, Diptera, Hemiptera and Coleoptera (Table 1 and 2; Appendices A, B, and C). The method used to collect these arthropods tended to capture small (~ 2.0 mm) ground dwelling or weak flying species. Because of its noise and limited suction capacity, the insect vacuum scared away or otherwise was unable to capture larger specimens, thus underestimating their abundance and diversity. However, the species collected were numerous and known residents in that community.

**Table 1:**
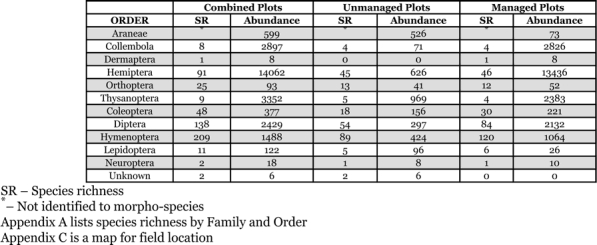
Species richness and abundance of arthropod orders according to field and management type

**Table 2:**
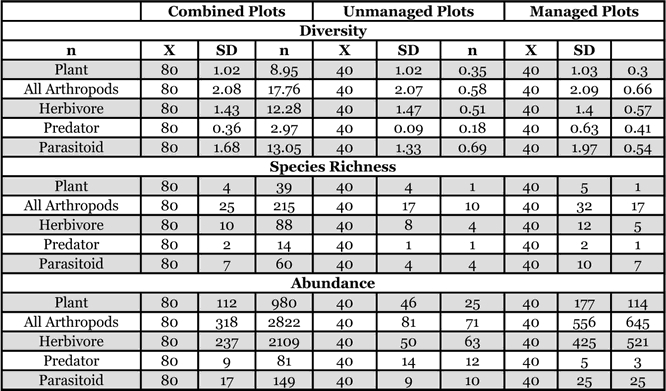


Total arthropod species richness for all plots was 267 ± 0.16 (SE), with a Chao 1 estimate of 341 ± 0.47 species. In the unmanaged fields, 174 ± 0.16 species were sampled, with a Chao 1 estimate of 247 ± 0.52. In the managed fields 214 ± 0.14 species were sampled, with a Chao 1 estimate of 266 ± 0.38 species, as much as the combined number of observed species for both management types.

**Figure 2:**
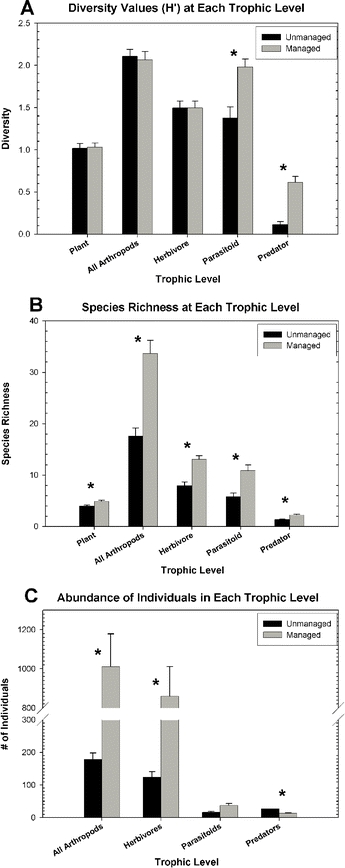
Shannon-Weiner Index (H’) (A), species richness (B), and abundance (C) for each trophic guild. Asterisks (*) indicate significant differences between management types (P_1,79_ < 0.0001). Grey bars = managed; black bars = unmanaged.

For both field types, the highest species richness was found in Hymenoptera, Diptera, Hemiptera, and Coleoptera, respectively. For the managed fields, the ranked order of dominance, based on abundance (not including Collembolans) was: Hemiptera, Thysanoptera, Diptera, Hymenoptera and Coleoptera, while for the unmanaged fields, the ranked order of dominance was: Thysanoptera, Hemiptera, Araneae, Hymenoptera and Diptera (Table 1). Managed fields had significantly higher plant abundance (F_[1,79]_ = 50.3, P < 0.0001; r^2^ = 0.39) and richness (F_[1,79]_ = 9.5, P = 0.003; r^2^ = 0.11) as well as overall arthropod abundance (F_[1,79]_ = 25.0, P < 0.0001; r^2^ = 0.25) and richness (F_[1,79]_ = 29.6, P < 0.0001; r^2^ = 0.28; Table 2 and 3). Managed fields were generally more diverse (except plant and herbivore diversity), had higher species richness and had greater abundance of all trophic groups, except predators (Fig 2) (spiders, the dominant predator, had very low abundance in managed fields). Overlap of shared species between managed versus unmanaged plot was low (Jaccard’s Similarity Index for all plots was 14.7 ± 0.47 SE, for unmanaged plots; 15.3 ± 0.67 SE, and managed plots; 21.0 ± .68 SE).

**Table 3:**
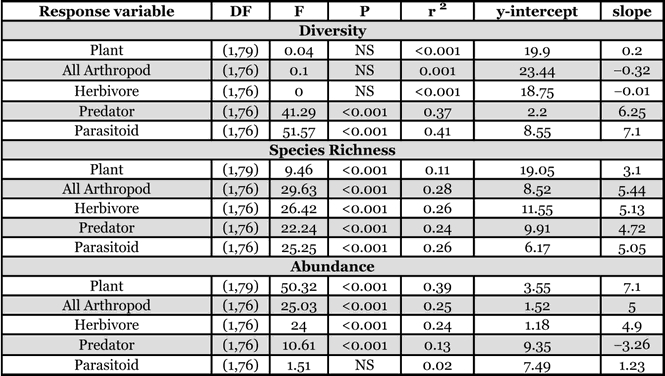
Parameter estimates and hypothesis tests for regressions of diversity variables on management type.

**Figure 3:**
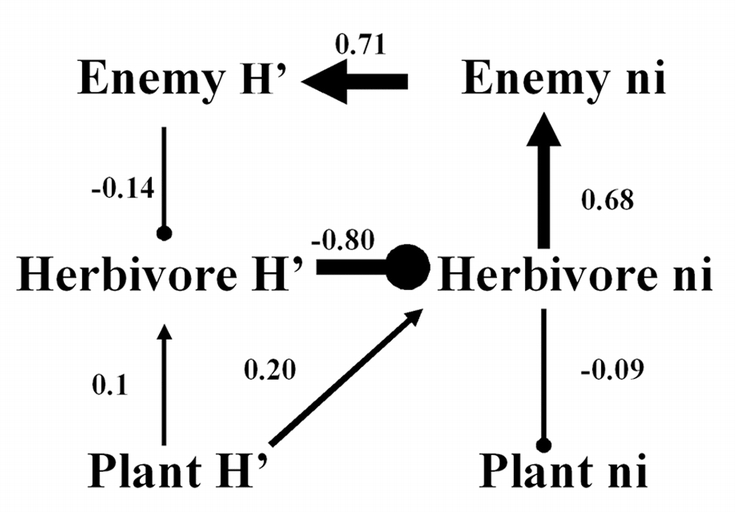
Path diagram based on analysis of data from managed plots. Solid arrows indicate direct positive effects, solid lines with a circle-head indicate direct negative effects. Numbers next to effects are values of the significant path coefficients. Thickness of lines indicates relative effect size. The model statistically fit the data (i.e. no significant differences between the correlational matrices of data versus model matrices; *X*^2^ = 12.55, DF = 8, P = 0.1281).

**Figure 4:**
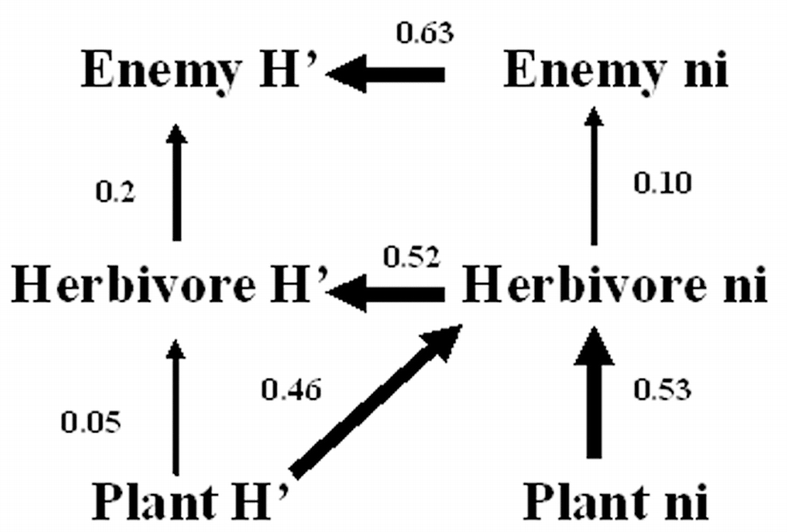
Path diagram based on analysis of data from unmanaged plots. Solid arrows indicate direct positive effects, solid lines with a circle-head indicate direct negative effects. Numbers next to effects are values of the significant path coefficients. Thickness of lines indicates relative effect size. The model statistically fit the data (*X*^2^ = 11.11, DF = 8, P = 0.1951).

Path analyses revealed strong effects of herbivore diversity on diversity of other trophic levels. For the managed fields (higher enemy diversity), our data supported the causal hypothesis that enemy diversity depressed herbivore diversity, which in turn depressed herbivore abundance, then plant abundance (Fig. 3). For the unmanaged fields (lower overall richness of plants and arthropods), the data supported the hypothesis that plant diversity caused increased herbivore diversity, which in turn increased enemy diversity (Fig. 4).

## Discussion

The two ecosystems examined in this study are different in most of the characteristics measured, and this was reflected in the interactions between diversities at different trophic levels. The managed fields are grazed at intervals by cattle and are periodically flood irrigated. These fields are highly productive, resource rich (water and nitrogen input from irrigation and cattle) environments that experience a fair amount of disturbance (flooding and grazing) throughout the growing season. It is possible that the increase in resources and mild disturbance regime account for the general increase in diversity across trophic levels in the managed fields. In contrast, the unmanaged fields are fenced to keep cattle out and are usually undisturbed, except for a limited weed control program. No herbicide spraying occurred near these plots at the time of sampling or during that growing season. Protected fields are less productive and support no cattle (elk and deer are the other large vertebrate herbivores). The only water source is from the water table and precipitation. These fields also had higher standing plant biomass and had a decomposing litter layer (personal observation).

We cannot generalize about the differences noted here between managed and unmanaged fields because of the absence of replicated treatments. Furthermore, without experimental manipulations or greater variation in management from plot to plot, it is difficult to determine which factors were responsible for the differences in plant and arthropod diversity between the two management types. The level of herbivory by cattle was not quantified, but considerable cattle impacts on vegetation were visible to the eye. It is likely that water inputs, nutrient enhancement, and cattle impact all contributed significantly to the major differences between management types. The most interesting difference between these two sites was the change in the strength of top-down versus bottom-up effects on plants and arthropods, with top-down forces being more important for plots in the generally more diverse managed fields, and bottom-up forces dominating the less diverse plots found in the unmanaged fields. This result is consistent with recent ideas that increases in overall diversity contribute to more effective control of herbivores by natural enemies (reviewed by Stireman et al. 2005). Since the managed, high diversity plots, had high resource input (water and nutrients), the results are also consistent with the “Ecosystem Exploitation Hypothesis” (Oksanen et al. 1981), which posits that higher resource availability allows for more robust upper trophic levels, enhancing top-down control.

The indirect effects of plant or enemy diversity on other trophic levels that was found for both management types support hypotheses from other empirical studies that have documented diversity cascades. Mechanisms for bottom-up diversity cascades are well described elsewhere (e.g., Siemann 1998) and suggest that increased productivity of plants causes greater plant diversity and creates greater habitat complexity, greater resource availability for high numbers of specialists, and other diversity enhancing factors. The top-down diversity cascades are less straightforward. Increased enemy abundance can cause both increases (e.g., Paine 1966) and decreases (e.g., Dyer and Letourneau 2003) in herbivore diversity, depending on the competitive position of the species most affected by changes in top-down mortality. Changes in enemy diversity can create the same patterns by affecting the abundance of enemies that regulate superior competitors. Increases in diversity of consumers are often accompanied by lower overall consumption (Schmitz et al. 2000), and less efficient regulation (Finke and Denno 2004). Thus, in the managed fields, the decrease in herbivore diversity putatively caused by decreases in enemy diversity could be due to depressed numbers of the enemy of the dominant herbivore competitor. Parasitoids were the most important guild of enemies in the managed plots, and elimination of species within any parasitoid family could certainly cause the high abundances of the numerically dominant cicadellids and thrips. It is also quite possible that in these managed plots, cattle grazing made herbivores more susceptible to enemy effects, due to depletion of available plant tissue and direct consumption of the herbivores by cattle.

The patterns uncovered in this study are the basis for current experimental tests of diversity cascades, and offer a relatively clear picture of trophic diversity relationships in these two different communities. The species richness of arthropods in these systems is quite high (350 total species estimated). The dominant orders collected were Diptera, Hymenoptera, Hemiptera and Coleoptera, and these accounted for over 75% of the sampled community (both in species richness and abundance). Almost 90% of individual hymenopterans collected were parasitoids (see appendix B). The vacuum sampled a subset of the community as it under-sampled the larger arthropods, but any single method of collecting arthropods is bound to be an underestimate of species present, and the smaller arthropods collected in our study are often under-sampled using techniques such as sweep-nets and pitfall traps. Our method focused on arthropods of a similar size range, and the species collected represent a majority of the diversity in the field ecosystems sampled, sampling about 80% of the estimated species richness.

Results from empirical diversity studies are likely to depend on conditions of the ecosystem under study. Our results from grasslands in Colorado are no exception. However, as more studies accumulate on interactions between abundance and diversity at different trophic levels, generalities will surely emerge. The results here are consistent with the idea that as overall diversity increases natural enemies have stronger effects on biotic communities, while less diverse communities are dominated by the effects of plant resources. As entomologists document abundance and richness of arthropods across broader diversity gradients, it will be interesting to see if this trend extends into higher levels of diversity, or if the relative magnitude of top-down and bottom-up effects cycles along a broad gradient of diversity.

## Figures and Tables

**Appendix A:**
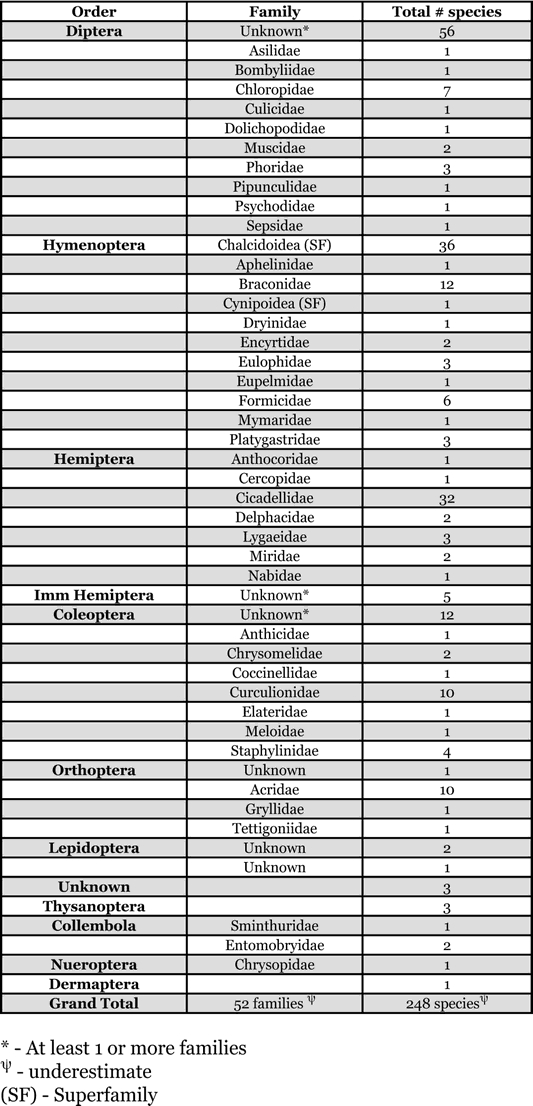
List of major Arthropods found in all plots Taxonomic list of arthropods found in all plots and is a pooling of both community types. List is organized based on species richness.

**Appendix B:**
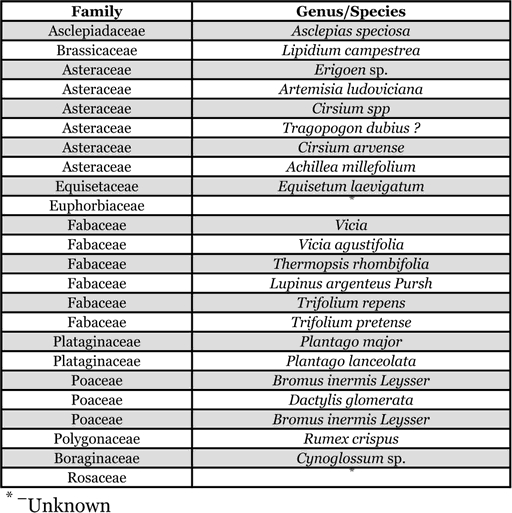
Taxonomic list of the major plants found in all plots

**Appendix C:**
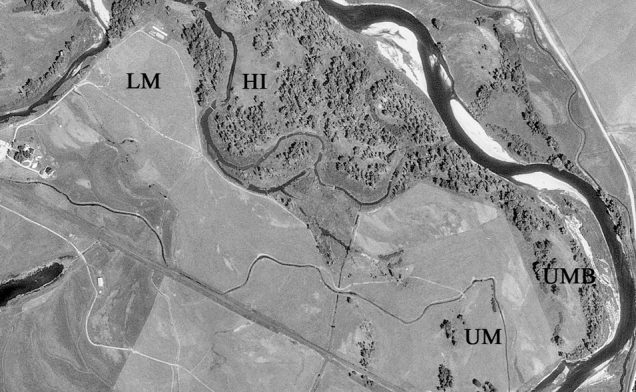
Aerial photograph showing location and type of field. North is the top of the picture. HI & UMB = unmanaged fields LM & UM - managed fields
